# Learning Curve and Clinical Outcomes of Ultrasonic Osteotome‐based En Bloc Laminectomy for Thoracic Ossification of the Ligamentum Flavum

**DOI:** 10.1111/os.13804

**Published:** 2023-07-05

**Authors:** Yuanyu Hu, Yanlei Dong, Junbo Qi, Zhongqiang Chen, Weishi Li, Yun Tian, Chuiguo Sun

**Affiliations:** ^1^ Department of Orthopaedics Peking University Third Hospital Beijing China; ^2^ Engineering Research Center of Bone and Joint Precision Medicine Beijing China; ^3^ Beijing Key Laboratory of Spinal Disease Research Beijing China

**Keywords:** En Bloc Laminectomy, Learning Curve, Thoracic Ossification of the Ligamentum Flavum, Ultrasonic Osteotome

## Abstract

**Objective:**

Despite rapid advances in minimally invasive surgery, en bloc laminectomy remains the most common surgical approach for treating thoracic ossification of the ligamentum flavum (TOLF). However, the learning curve of this risky operation is rarely reported. Therefore, we aimed to describe and analyze the learning curve of ultrasonic osteotome‐based en bloc laminectomy for TOLF.

**Methods:**

Among 151 consecutive patients with TOLF who underwent en bloc laminectomy performed by one surgeon between January 2012 and December 2017, we retrospectively analyzed their demographic data, surgical parameters, and neurological function. Neurological outcome was evaluated with the modified Japanese Orthopaedic Association (mJOA) scale, and the Hirabayashi method was used to calculate the neurological recovery rate. The learning curve was assessed with logarithmic curve‐fitting regression analysis. Univariate analysis methods were used for statistical analysis, including *t*‐test, rank sum test, and chi‐square test.

**Results:**

A total of 50% of learning milestones could be reached in approximately 14 cases, and the asymptote in 76 cases. Therefore, 76 of the 151 enrolled patients were defined as the “early group,” and the remaining 75 were delimitated as the “late group” for comparison. There was a significant intergroup difference in the corrected operative time (94.80 ± 27.77 *vs* 65.93 ± 15.67 min, *P* < 0.001) and the estimated blood loss (median 240 *vs* 400 mL, *P* < 0.001). The overall follow‐up was 83.1 ± 18.5 months. The mJOA significantly increased from a median of 5 (IQR: 4–5) before the surgery to 10 (IQR: 9–10) at the last follow‐up (*P* < 0.001). The overall complication rate was 37.1%, and no significant intergroup difference was found, except for the incidence of dural tears (31.6% *vs* 17.3%, *p* = 0.042).

**Conclusion:**

Initially, mastering the en bloc laminectomy technique using ultrasonic osteotome for TOLF treatment can be challenging, but the surgeon's experience improves as the operative time and blood loss decrease. Improved surgical experience reduced the risk of dural tears but was not associated with the overall complication rate or long‐term neurological function. Despite the relatively long learning curve, en bloc laminectomy is a secure and valid technique for TOLF treatment.

## Graphical abstract

En bloc laminectomy has been the standard surgical technique for treating thoracic ossification of the ligamentum flavum (TOLF). We described and analyzed the learning curve of ultrasonic osteotome‐based en bloc laminectomy for TOLF. The results indicated that the mastery of this surgical technique could be challenging initially, but the surgeon's experience improved as the operative time and blood loss decreased. Improved surgical experience reduced the risk of dural tears but was not associated with the overall complication rate or long‐term neurological function. Despite the relatively long learning curve, en bloc laminectomy is a secure and valid technique for TOLF treatment.

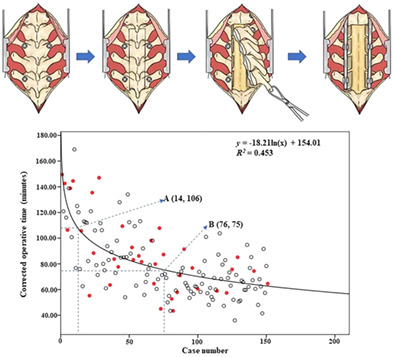

## Introduction

Thoracic spinal stenosis is a relatively rare but severe condition that can result in neurological deterioration, and one of its main etiologies is thoracic ossification of the ligamentum flavum (TOLF).^1^, ^2^, ^3^ When spinal cord compression‐related symptoms progressively aggravate, surgical treatment is the primary option for neurological recovery.^4^, ^5^ En bloc laminectomy, including the resection of the lamina and ossified ligamentum flavum, is the most common operation for TOLF.^6^, ^7^, ^8^ Compared to piecemeal laminectomy, the en bloc method has multiple advantages, including reduced operative time, blood loss, and risk of spinal cord or nerve root injury.^7^, ^9^, ^10^ Thoracic spine surgery using ultrasonic osteotomes has been proven secure and valid compared to high‐speed drills; additionally, it has been shown to significantly reduce surgical time and blood loss.^6^, ^11^, ^12^ Given these advantages, the combination of en bloc laminectomy and ultrasonic osteotome will hopefully sufficiently bring the benefits of both methods into play.

However, performing en bloc laminectomy with an ultrasonic osteotome is challenging because of the nature of the disease and the unfamiliar technical characteristics.^6^, ^12^ First, the blood supply to the thoracic spinal cord is fragile, and the risk of postoperative complications is high.^3^, ^13^, ^14^ Second, dural ossification and adhesion occur in 25% and 35% of patients with TOLF, respectively, which increases the surgical difficulty and the risk of iatrogenic dura tears and nerve injury.^15^, ^16^, ^17^, ^18^ Third, due to the lack of direct visualization and anatomic reference of the dura, the blade may accidentally be inserted into the spinal canal when cutting the laminae with an ultrasonic osteotome, thus injuring the spinal cord or nerve roots.^19^ A learning curve is essential to evaluate the surgeon's ability and help avoid severe complications and repeated mistakes when using the technique initially, especially for complicated and risky procedures.^20^, ^21^, ^22^ Multiple research groups have described and analyzed the learning curve of navigated spinal deformity correction and minimally invasive spinal surgery, which helps to understand and disseminate these new technologies.^22^, ^23^, ^24^, ^25^

However, to our knowledge, no studies have described the learning curve for en bloc laminectomy in treating TOLF. This may be due to the low disease incidence, making it difficult for a single surgeon to accumulate enough experience to evaluate the learning effect quickly. Our center is one of the largest thoracic spine surgery centers in China, and the senior author who specialized in this area has already performed over 600 thoracic spine surgeries in the past decade. Therefore, we can conduct research on the learning curve for en bloc laminectomy in treating TOLF. Herein, we aimed to: (i) construct the learning curve of en bloc laminectomy using ultrasonic osteotome; (ii) analyze and compare the short‐term and long‐term surgical outcomes of surgeries at different periods; and (iii) offer some useful tips for inexperienced surgeons.

## Materials and Methods

### Patient Population

**Fig. 1:**
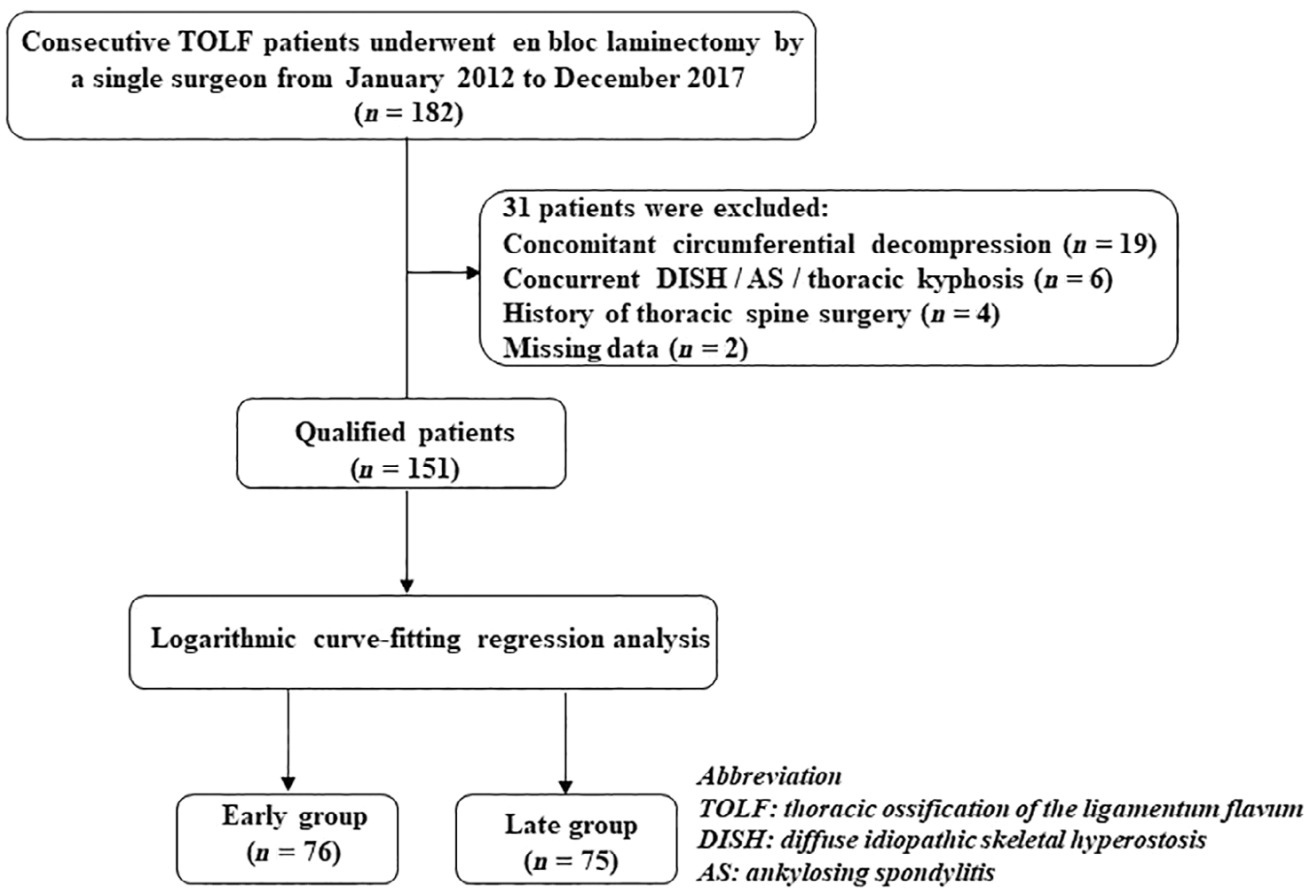
Flow chart of this study based on the STROBE statement.

The data of 182 patients who underwent en bloc laminectomy for TOLF between January 2012 and December 2017 were collected and analyzed. The same senior surgeon who had not completed the procedure independently but had been trained as an assistant in more than 50 cases of thoracic laminectomy performed all surgeries. The inclusion criteria were the presence of radiographic evidence and myelopathy symptoms of TOLF and en bloc laminectomy using an ultrasonic osteotome. Patients with a history of thoracic spine surgery, diffuse idiopathic skeletal hyperostosis, ankylosing spondylitis, or concomitant circumferential decompression were excluded. The patients who underwent en bloc laminectomy and concomitant circumferential decompression were excluded because of the possibility of bias induced by the following factors: First, circumferential decompression is performed when ventral compression to the spinal cord is evident, which is different from most TOLF with simple posterior compression in this study. Second, as a riskier procedure, circumferential decompression was not allowed to be performed until the senior surgeon had completed 40 en bloc laminectomies independently in the 4th year, which may significantly interfere with the learning curve. The inclusion and exclusion criteria and details of the study selection process are presented in Fig. 1. This study was approved by our institution's Medical Science Research Ethics Committee (No. M2022083). Informed consent was not required because of the retrospective nature of the study.

### Surgical Technique

**Fig. 2:**
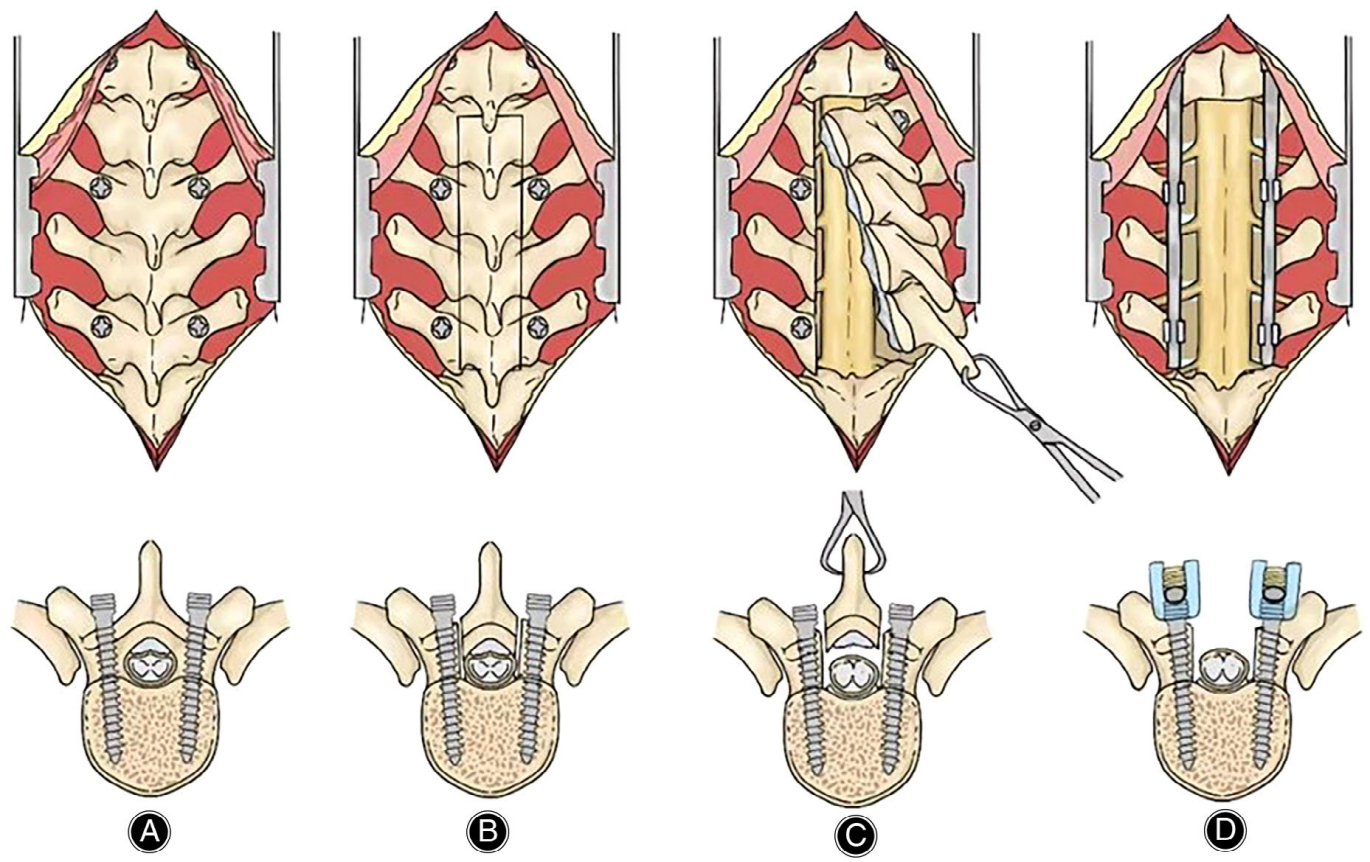
Schematic diagrams of en bloc laminectomy for ossification of ligamentum flavum with ultrasonic bone scalpel. (A) Insert pedicle screw after exposing the lamina and transverse process; (B) Cut through the lamina and ossified ligament along the midline of bilateral facet joints; (C) Separate the ossified ligament and the dura matter; (D) Pressurize fixation with screw‐rod system.

This surgical technique has been demonstrated previously.^6^ All procedures were conducted with the patient under endotracheal intubation general anesthesia in a prone position. Fluoroscopic imaging was used to identify the specific spine segment that needed surgical attention. The surgeon then made a posterior midline incision to access the transverse processes and lamina of the targeted segment. Subsequently, the surgeon removed various ligaments and bone structures, including the dorsal spinous process, supraspinatus ligament, and interspinous ligament, using rongeurs. Pedicle screws were inserted as positioning markers before laminectomy. The surgeon cut the lamina longitudinally along the midline of the facet joints using the ultrasonic osteotome XD860A (SMTP Technology Co., Ltd. Beijing, China). This process was finished when the entire layer of the bone structure was cut through. Subsequently, the surgeon gradually lifted the lamina with towel forceps while using a nerve stripper to separate the ossified ligament and dural. En bloc laminectomy of the lamina and ossified ligaments was completed when the lamina was fully lifted and the screw‐rod system was secured (Fig. 2).

### Outcome Evaluation and Statistical Analysis

The perioperative parameters and complications were analyzed. Neurological outcomes were assessed with the modified Japanese Orthopaedic Association score (mJOA, total score of 11 points) at the routine 3‐month follow‐up and yearly after that. Notably, the operative time strongly correlates with the number of decompression levels. Therefore, we calculated the corrected operative time to eliminate the influence of the number of decompression levels. The process for obtaining the corrected operative time involved grouping patients into seven categories based on the number of decompressed levels and calculating the average operative duration for each group. The mean operating time of each group was divided by that of the two‐level group to calculate the operative time coefficient for each group. The corrected operative time was obtained through the operative time for each patient divided by the corresponding coefficient. The coefficients of the three‐, four‐, five‐, six‐, seven‐, and more than seven‐level groups are 1.29, 1.52, 1.75, 2.02, 2.11, and 2.73, respectively (Table 1). Statistical analysis was performed using SPSS (Version 27.0, Armonk, NY, USA), and significance was set at *P* < 0.05. The Shapiro–Wilk test was used to check the normality of the data. Continuous data were expressed as mean and standard deviation or interquartile range. Continuous data with and without normality were analyzed using the *t*‐test and Mann–Whitney U test, respectively. Repeated‐measures data were compared with the Friedman test. Categorical data between the early and late groups were compared with the chi‐square test or Fisher's exact test. A logarithmic curve‐fitting regression analysis was applied to evaluate the en bloc laminectomy learning curve for TOLF.

**TABLE 1:** Comparison of the mean operative time between groups by number of decompressed levels

Decompressed levels	Number of cases	Operative time (min)	Coefficient *K*
2	37	80.89 ± 26.77	1.00
3	59	100.32 ± 32.28	1.29
4	29	121.10 ± 43.93	1.52
5	10	145.10 ± 45.25	1.75
6	6	204.50 ± 76.18	2.02
7	5	194.60 ± 71.78	2.11
≥8	5	220.60 ± 49.24	2.73

*Note*: Coefficient *K* = mean operative time of *n*‐level surgery/mean operative time of 2‐level surgery.

## Results

### General Data

**Fig. 3:**
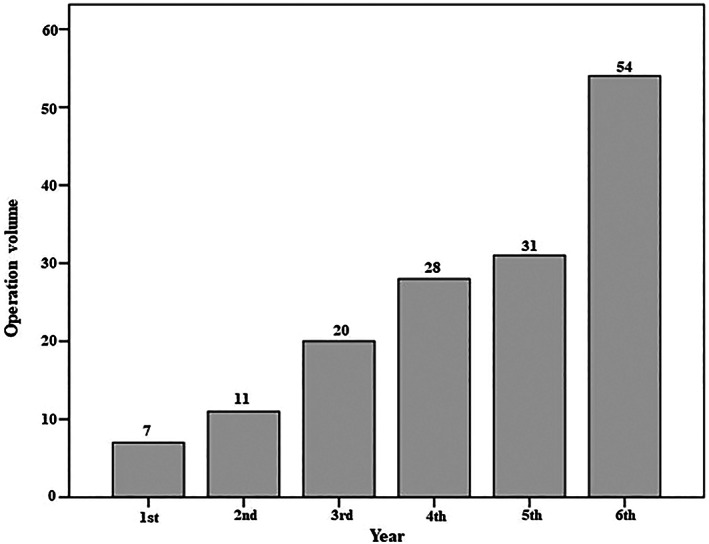
Bar graph showing the number of procedures performed yearly.

**Fig. 4:**
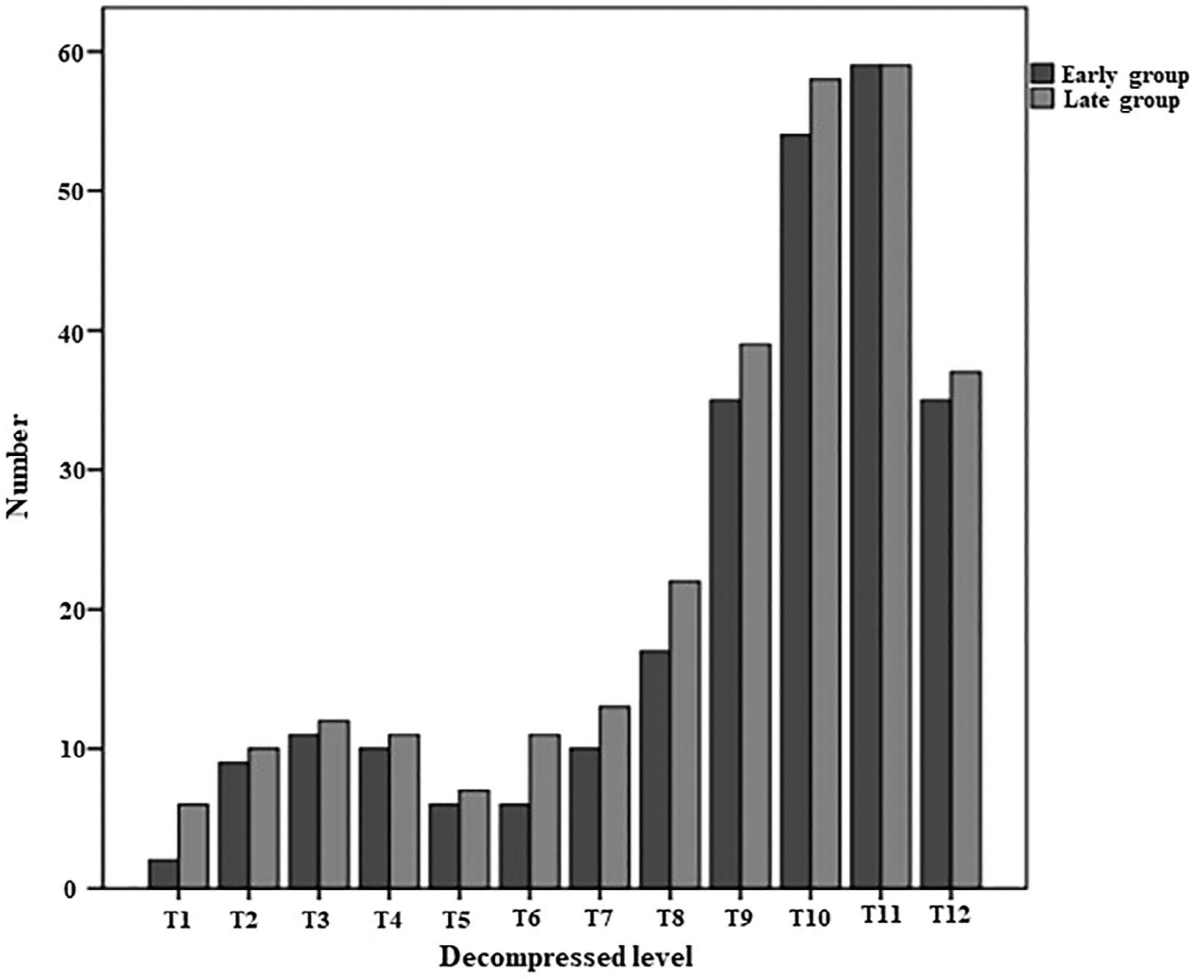
Bar graph showing the distribution of the decompressed segments.

As shown in Table 2, 151 TOLF patients with complete clinical data (56.4 ± 9.6 years, 53.0% male) were included. The operation volume increased yearly, with only seven surgeries performed in the first year and 54 in the 6th year (Fig. 3). The median age‐adjusted Charlson Comorbidity Index was 3, and the median symptom duration was 6 months. Most patients (82.1%) had only TOLF as a pathological diagnosis, whereas only 17.9% had concurrent ossification of the posterior longitudinal ligament or thoracic disc herniation. In total, 125 patients (82.8%) had no more than four operated segments. Figure 4 shows the distribution of the decompressed levels, which is consistent with a previously reported conclusion that TOLF is mainly distributed in the lower thoracic spine.^1^, ^8^, ^12^ No statistically significant difference was found in the distribution of the decompressed segments between groups. The mean operative time of all patients was 111.7 ± 50.6 min, and the median intraoperative blood loss was 300 (IQR 200–500) mL.

**TABLE 2:** Demographic and surgical characteristics of 151 patients

Characteristics	Total (*n* = 151)
Age (years)	56.35 ± 9.56
Male (*n*, %)	80 (53.0%)
BMI (kg/m^2^)	26.88 ± 4.30
Smoking (*n*, %)	11 (7.3%)
Hypertension (*n*, %)	67 (44.4%)
Diabetes Mellitus (*n*, %)	20 (13.2%)
ACCI	3 (2–4)
Duration of symptoms (month)	6 (2–12)
Preoperative functional status	
Independent (*n*, %)	88 (58.3%)
Partially dependent (*n*, %)	46 (30.4%)
Totally dependent (*n*, %)	17 (11.3%)
Etiological diagnosis (*n*, %)	
OLF only	124 (82.1%)
OLF with OPLL	23 (15.2%)
OLF with TDH	4 (2.7%)
Number of decompressed levels	3.58 ± 1.78
1–4 levels (*n*, %)	125 (82.8%)
5–8 levels (*n*, %)	22 (14.6%)
9–12 levels (*n*, %)	4 (2.6%)
Operative time (min)	111.73 ± 50.60
Estimated blood loss (mL)	300 (200–500)
Hospital stay (day)	8 (7–10)
Follow‐up (month)	83.12 ± 18.53

*Note*: Values are presented as number of cases (%), mean ± SD, or median (IQR).

Abbreviations: ACCI, age‐adjusted Charlson Comorbidity Index; BMI, body mass index; OLF, ossification of ligamentum flavum; OPLL, ossification of posterior longitudinal ligament; TDH, thoracic disc herniation.

### Learning Curve for En Bloc Laminectomy with Ultrasonic Osteotome

**Fig. 5:**
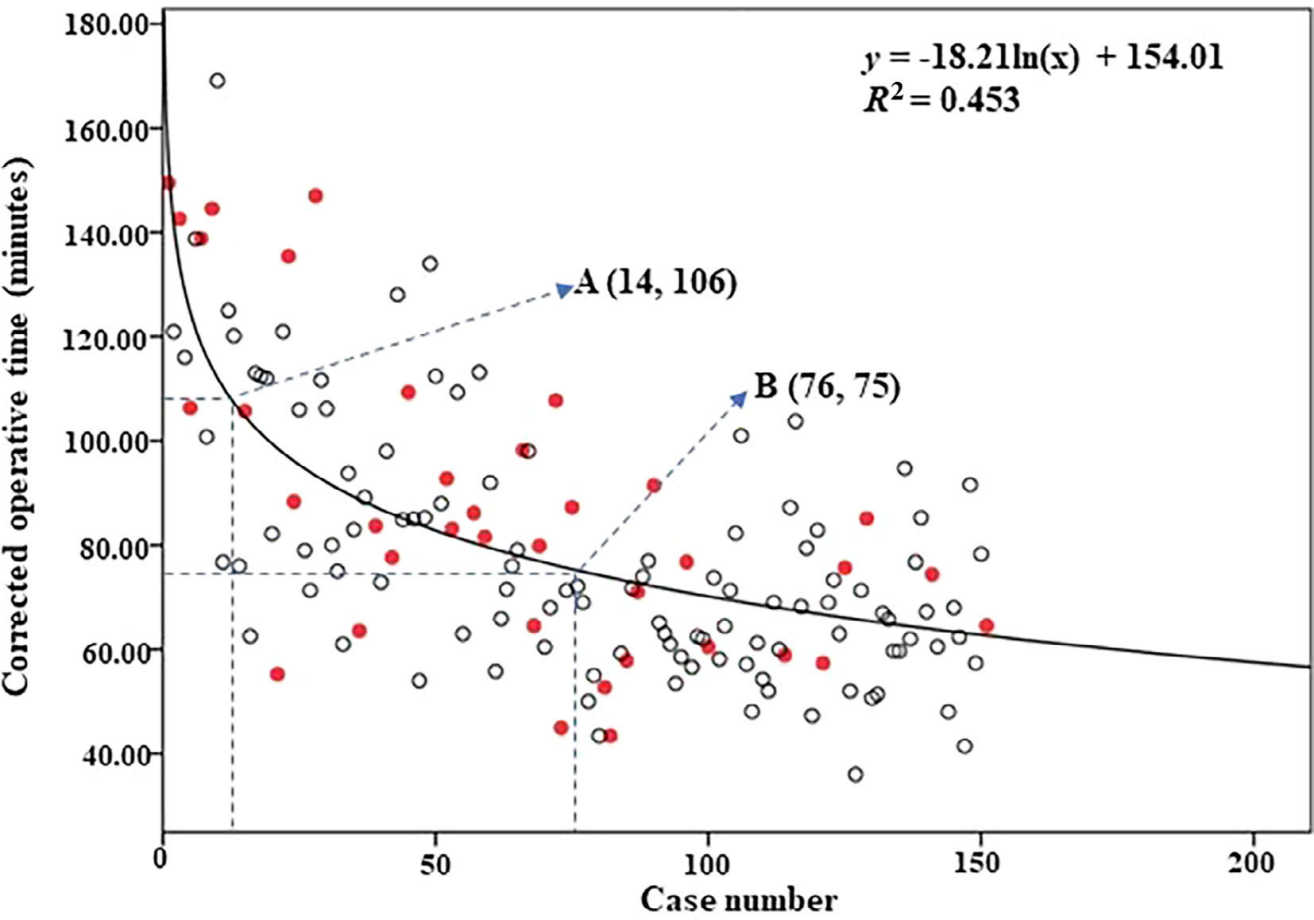
Learning curve for en bloc laminectomy as shown by corrected operative time: Distribution of dural tear (red spot); 50% Learning milestone (A); Asymptote (B).

As shown in Fig. 5, as the surgeon gained more experience, the corrected operative time decreased, which followed the pattern that was demonstrated by the function obtained from the logarithmic curve‐fitting analysis ($y=-18.21\ln\left(x\right)+154$) with a coefficient of determination *R* ^2^ of 0.453 (case number, *x*; corrected operative time, *y*(min)). The learning curve was steep in the initial stage, and 50% improvement was expected after 14 cases. However, the learning curve did not reach a stable plateau until approximately 76 cases were operated on.

### Surgical Parameters

Based on the learning curve, 151 patients were divided into the early (76 patients) and late (75 patients) groups, respectively. No statistical difference was found in the demographic characteristics between groups (Table 3). However, surgical parameters varied significantly between groups. A significant decrease in the corrected operative time was observed between the early (94.80 ± 27.77 min) and the late group (65.93 ± 15.67 min) (*P* < 0.001). A significant reduction was found in estimated blood loss from 400 (IQR, 270–600) to 240 mL (IQR, 150–300) (*P* < 0.001). Additionally, the drainage time decreased from 4.5 (IQR, 4.0–5.0) to 4.0 days (IQR, 3.0–4.0) (*p* < 0.001), and the hospital stay decreased from 9 (IQR, 7–11) to 8 days (IQR, 7–9) (*P* = 0.034).

**TABLE 3:** Intergroup comparison of characteristics for two groups

Characteristics	Early group (*n* = 76)	Late group (*n* = 75)	Statistic value^a^	*P* value
Age (years)	56.13 ± 9.61 (34–81)	56.57 ± 9.57 (33–78)	−0.282	0.778
Sex, male (*n*, %)	40 (52.6%)	40 (53.3%)	0.007	0.931
BMI (kg/m^2^)	26.85 ± 4.22	26.89 ± 4.39	0.057	0.957
Duration of symptoms (month)	6 (3–12)	4 (2–12)	−0.609	0.543
Preoperative functional status				
Independent (*n*, %)	49 (64.5%)	39 27 (52.0%)	2.416	0.120
Partially dependent (*n*, %)	19 (25.0%)	27 (36.0%)	2.156	0.142
Totally dependent (*n*, %)	8 (10.5%)	9 (12.0%)	0.082	0.775
Hypertension (*n*, %)	29 (38.2%)	38 (50.7%)	2.393	0.122
Diabetes Mellitus (*n*, %)	10 (13.2%)	10 (13.3%)	0.001	0.975
ACCI	3 (2–4)	3 (2–4)	−1.223	0.221
Etiological diagnosis (*n*, %)				
OLF only	63 (82.9%)	61 (81.3%)	0.063	0.802
OLF with OPLL	11 (14.5%)	12 (16.0%)	0.068	0.794
OLF with TDH	2 (2.6%)	2 (2.7%)	‐	0.989
Number of decompressed levels	3.34 ± 1.66	3.81 ± 1.86	−1.639	0.103
Dural adhesion (*n*, %)	26 (32.9%)	21 (29.3%)	0.679	0.636
Operative time (min)	126.00 ± 55.12	97.84 ± 41.65	3.545	0.001
Corrected operative time (min)	94.80 ± 27.77	65.93 ± 15.67	7.881	<0.001
Estimated blood loss (mL)	400 (270–600)	240 (150–300)	−4.726	<0.001
Blood transfusion (*n*, %)	8 (10.5%)	3 (4.0%)	2.380	0.123
Drainage time (day)	4.5 (4.0–5.0)	4.0 (3.0–4.0)	−4.671	<0.001
Hospital stay (day)	9 (7–11)	8 (7–9)	−2.119	0.034

*Note*: Values are presented as number of cases (%), mean ± SD, or median (IQR).

Abbreviations: ACCI, age‐adjusted Charlson Comorbidity Index; CSFL, cerebrospinal fluid leakag; OLF, ossification of ligamentum flavum; OPLL, ossification of posterior longitudinal ligament; TDH, thoracic disc herniation.ACCI: age‐adjusted Charlson Comorbidity Index; OLF: ossification of ligamentum flavum; OPLL: ossification of posterior longitudinal ligament; TDH: thoracic disc herniation; CSFL: cerebrospinal fluid leakage.

^a^ For *t*‐test, the statistic value refers to *t* value; For *χ* ^2^ test, the statistic value refers to *χ* ^2^ value; For Mann–Whitney *U* test, the statistic value refers to *Z* value; For Fisher's exact test, there is no statistic value.

### Surgical Complications

As shown in Table 4, out of 151 cases, 56 experienced complications, resulting in a total complication rate of 37.1%. There was no statistical difference in complication rate between groups (*P* = 0.199). However, the late group had a lower incidence of intraoperative dural tears than did the early one (17.3% *vs* 31.6%, *P* = 0.042), and the distribution of dural tears is also demonstrated in Fig. 5. The rates of cerebrospinal fluid leakage in the early and late groups were 38.2% (29/76) and 26.7% (20/75), respectively. Although the rate was greater in the early group, no significant difference was found (*P* = 0.132). Apart from the cerebrospinal fluid leakage, the complication rate was 4.6% (7/151). Seven critical complications required unplanned reoperation: cerebrospinal fluid pseudocyst (*n* = 2), wound dehiscence (*n* = 2), wound infection (*n* = 1), hematoma (*n* = 1), and inadequate decompression (*n* = 1). All seven cases improved after reoperation without serious sequelae. There were three cases of nerve root injury and transient nerve deterioration, all of which improved after conservative treatment. In these cases, two nerve root injuries occurred when the blade of the ultrasound bone scalpel cut through the lamina. No iatrogenic spinal cord injury was observed intraoperatively.

**TABLE 4:** Summary of perioperative complications (*n* = 151)

Complications	Early group (*n* = 76)	Late group (*n* = 75)	Statistical value^a^	*P* value
Total complications (*n*, %)	32 (42.1%)	24 (32.0%)	1.652	0.199
Dural tear (*n*, %)	24 (31.6%)	13 (17.3%)	3.407	0.042
CSFL (*n*, %)	29 (38.2%)	20 (26.7%)	2.274	0.132
Pseudocyst (case NO.)	3 (6, 27, 43)	2 (99, 126)	‐	0.506
Wound dehiscence (case NO.)	1 (49)	1 (129)	‐	1.000
Surgical site infection (case NO.)	1 (2)	0 (−)	‐	1.000
Hematoma (case NO.)	1 (59)	0 (−)	‐	1.000
Inadequate decompression (case NO.)	1 (19)	0 (−)	‐	1.000
Nerve root injury (case NO.)	2 (11, 38)	1 (114)	‐	0.568
Neurological deterioration (case NO.)	1 (15)	2 (137,149)	‐	0.620
Requiring unplanned reoperation (*n*, %)	4 (5.3%)	3 (4.0%)	‐	1.000

*Note*: Values are presented as number of cases (%) or number of cases (case NO.).

Abbreviation: CSFL: cerebrospinal fluid leakage.

^a^ For *χ* ^2^ test, the statistic value refers to *χ* ^2^ value; For Fisher's exact test, there is no statistic value.

### Neurological Outcomes

The mean duration of follow‐up was 83.1 ± 18.5 months. Throughout the follow‐up, a total of 13 patients (8.6%) were lost at various time points, with eight from the early group and five from the late one. As a result, neurological function data from the last follow‐up were used for statistical analysis. The mJOA scores of all patients significantly increased from five points (IQR, 4–5) before surgery to eight points (IQR, 7–8) 3 months after surgery (*P* < 0.001) and 10 points (IQR, 9–10) at last follow‐up (*P* < 0.001). The median recovery rate of the last follow‐up in the early and late groups was 80.0% (IQR, 62.5%–88.5%) and 81.3% (IQR, 66.7%–85.7%), respectively. Notably, the two groups had no significant differences in the mJOA scores or recovery rate at the same time points (Table 5).

**TABLE 5:** Intergroup comparison of neurological outcomes for two groups

Characteristics	Early group (*n* = 76)	Late group (*n* = 75)	Statistical value^a^	*P* value
Follow‐up (months)	108.00 ± 14.30	68.00 ± 5.40	22.680	<0.001
mJOA				
Preoperative	5 (4–5)	5 (4–6)	−0.197	0.844
3‐month follow‐up	8 (7–9)	8 (7–8)	−0.156	0.876
Last follow‐up	10 (9–10)	10 (9–10)	−0.110	0.912
Recovery rate (%)				
3‐month follow‐up	50.00 (37.50–62.50)	50.00 (42.86–63.63)	−0.299	0.766
Last follow‐up	80.00 (62.50–88.54)	81.33 (66.67–85.71)	−0.135	0.895

*Note*: Recovery rate = $\frac{\text{postoperative mJOA}-\text{preoperative mJOA}}{11-\text{preoperative mJOA}}$. Values are presented as mean ± SD or median (IQR).

Abbreviation: mJOA: modified Japanese Orthopedic Association score.

^a^ For *t*‐test, the statistic value refers to *t* value; For Mann–Whitney *U* test, the statistic value refers to *Z* value.

## Discussion

In this study, we constructed a learning curve of en bloc laminectomy with an ultrasonic bone scalpel for TOLF, which might have a positive reference value for inexperienced surgeons. With 76 cases as the asymptote point, the intra‐operative parameters of the late group were significantly improved compared with the early group, but there was no statistical intergroup difference in surgery‐related complications and neurological outcomes.

### Learning Curve for Spine Surgery

Although endoscopic surgery has successfully treated isolated TOLF, en bloc laminectomy remains the standard procedure.^26^, ^27^ However, en bloc laminectomy remains challenging for inexperienced surgeons because of dural adhesion and ossification induced by TOLF.^4^, ^18^, ^28^ Moreover, the unfamiliar characteristics of the ultrasonic tools and limited surgical field vision increase the difficulty and risk of surgery.^29^ The great surgical difficulty and danger may discourage spine surgeons from learning and performing such surgery. Consequently, a proper estimation of the learning process of the technique is essential for inexperienced surgeons. The learning curve of ultrasonic osteotome‐based en bloc laminectomy represents the learning course and provides surgeons with an overview of the learning process. Following this strategy, a consecutive series of patients were used to mimic the learning process for all surgeons.

Logarithmic curve fitting regression has been the primary mathematical model for studying the learning curve in previous studies, and its effectiveness has been thoroughly proven.^21^, ^22^, ^23^, ^24^, ^25^, ^30^ The learning curve for minimally invasive lumbar surgery was reported by Yang *et al*.^23^, ^24^ and Silva *et al*., with *R* ^2^ values of 0.56 and 0.57, respectively. Bourghli *et al*.^20^ and Hyun *et al*.^21^ reported the learning curve of deformity correction, with an *R* ^2^ value of less than 0.3. Similarly, we mathematically derived various functions and proved that the logarithmic model had the largest *R* ^2^ value, indicating that it best explained the data. The *R* ^2^ value of this learning curve is 0.453, which is smaller than that of the minimally invasive lumbar surgery and significantly larger than that of deformity correction. This suggests that for complex procedures, the surgery duration depends not only on the surgeon's experience but is also influenced by other factors such as the assistant, scrub nurse, and fluoroscopy technician.^30^ In addition, en bloc laminectomy for TOLF was associated with more significant variation in surgical sites and number of segments than minimally invasive lumbar surgery. Therefore, the data remained heterogeneous even after correction, resulting in a smaller *R* ^2^.

Two studies reported the learning curve of minimally invasive lumbar fusion, which decreased significantly at early stages.^24^, ^30^ Our learning curve follows a similar trend at the early stage and reaches a 50% learning milestone in 14 cases. As for the asymptote, the learning curve of minimally invasive lumbar surgery was reported by several research groups, all of which reached the asymptote around 20 to 30 cases.^23^, ^24^, ^25^ The asymptote of the learning curve of our study was not reached until approximately 76 cases, which is much larger than that of the previous studies but comparable to that of the complicated scoliosis correction procedure.^20^ This suggests that the long learning curve may be associated with the complexity of the procedure. Besides, only 38 surgeries were performed in the first 3 years, impeding short‐term high‐density learning reinforcement for the surgeon.

### Short‐term and Long‐term Surgical Outcomes

Notably, the mean operative time of the whole cohort was 111.7 ± 50.60 min, comparable to the 125.8 min in previous studies using ultrasonic osteotome and much smaller than the 189.4–296 min using a high‐speed drill.^11^, ^12^ With the increased familiarity with the skills and knowledge of the pathological changes of TOLF, the mean corrected operative time successfully decreased from 94.80 min in the early group to 65.93 min in the late one. The median blood loss was 240 mL, lower than the 417 mL reported in the literature.^11^, ^12^ Unsurprisingly, the drainage time and hospital stay decreased as the surgical parameters improved.

A major concern for the patients and surgeons is complications during the initial stage of performing the en bloc laminectomy with an ultrasonic osteotome. Similar to those reported in the literature,^3^, ^16^, ^18^ the overall complication rate (37.1%) was high, but no difference was found between the two groups. Sun *et al*.^15^ and Ju *et al*.^16^ reported the incidence of dural laceration as 15% and 32%, respectively. Herein, the overall rate of dural tears and cerebrospinal fluid leakage was 24.5% and 32.5%, respectively, consistent with previous values. Due to the preoperative imaging evaluation of dural ossification and the improvements in surgical techniques, the incidence of intraoperative dural tears decreased significantly (31.6% *vs* 17.3%, *p* = 0.042). However, no statistical difference was observed between the cerebrospinal fluid leakage rates of the two groups (38.2% *vs* 26.7%, *p* = 0.132), which indicated that invisible intraoperative dural injury may still occur despite the decrease in the recorded rate of dural tears. Most patients with cerebrospinal fluid leakage recovered after conservative treatment, except for two patients with poor wound healing and one with a secondary surgical site infection. A total of seven patients (4.6%) required unplanned reoperation, which is also consistent with the revision rate of 3.3% to 4.0% reported previously.^14^, ^31^ There were three cases of transient neurological deterioration, all of which were recovered with conservative therapy. The lack of significant differences in complication rates between the two groups indicates the importance of consistently focusing on preventing complications, even after mastering the technique.

A significant improvement in mJOA scores was observed at the 3‐month milestone and last follow‐up compared to preoperative scores. This result further supports the efficacy of the surgical technique. Additionally, no significant difference in neurological outcomes was observed between the early and late groups at the same follow‐up time points, suggesting that similar long‐term results can be achieved regardless of experience level. Therefore, inexperienced surgeons should be encouraged to learn and master this technique despite more dural tears in the initial stage.

### Tips for Inexperienced Surgeons

The following three tips may help inexperienced surgeons shorten the learning curve for en bloc laminectomy and avoid serious complications. First, preoperative radiographic data should be carefully evaluated to discover the related manifestations of dural ossification, including tram track, comma, and banner signs, to prepare for intraoperative dural repair in advance.^16^, ^32^, ^33^ Second, pedicle screws, which can be used as anatomical markers to avoid accidental injury to the spinal cord, should be inserted before laminectomy. Third, it is recommended to retain the last thin layer of ossification rather than penetrate it and pry open the lamina with a narrow bone osteotome to avoid damage to the spinal cord and nerve roots by the ultrasonic osteotome.^6^

### Strengths and Limitations

Through long‐term follow‐up and comparative cohort study, the current study is the first to construct the learning curve of ultrasonic osteotome in treating TOLF, revealing considerable guiding significance for clinical work, especially for young doctors who learn to use an ultrasonic bone scalpel to treat TOLF.

This study had several limitations. First, the study's retrospective nature led to difficulties in study design and bias control. Second, follow‐up ended at different time points, which may have affected the interpretation of the neurological outcome. Third, although ultrasonic osteotome‐based en bloc laminectomy is only used for TOLF in our institution, laminectomy is also commonly used for cervical and lumbar spine disease, which may affect the learning curve.

### Conclusion

Based on a consecutive series of 151 patients with TOLF undergoing en bloc laminectomy with an ultrasonic osteotome, the current study first revealed the learning curve of this surgical technique and proved that a steady situation would be achieved after approximately 76 cases. The operative time, intraoperative blood loss, drainage time, and hospital stay improved as experience increased; however, no significant correlation was conducted between the perioperative complication rate, long‐term neurological function, and experience accumulation. These findings may have important value for surgeons at the initial stage of ultrasonic osteotome‐based en bloc laminectomy for TOLF.

## Author Contributions

Chuiguo Sun and Yun Tian provided the conception and designed this study. Yuanyu Hu and Yanlei Dong contributed to data collection and analysis. Junbo Qi, Zhongqiang Chen, and Weishi Li contributed to study guidance and manuscript revision. All authors participated in the surgical procedure and postoperative follow‐up.

## Funding Information

This work was supported by the Peking University Third Hospital Clinical Cohort Project (BYSYDL2022009, BYSYDL2021011).

## Conflict of Interest Statement

The authors have no further conflicts of interest to report.

## Ethics Statement

The study protocol was approved by the Medical Science Research Ethical Committee of Peking University Third Hospital (Ethics No.: M2022083). For this type of study, formal consent is not required. All procedures performed in studies involving human participants were in accordance with the ethical standards of the institutional and/or national research committee and with the 1964 Helsinki declaration and its later amendments or comparable ethical standards.

## Data Availability

The datasets generated during and/or analyzed during the current study are available from the corresponding author upon reasonable request.
